# The Influence of 1-Triacontanol on the Growth, Flowering, and Quality of Potted *Bougainvillea* Plants (*Bougainvillea glabra* var. “Elizabeth Angus”) under Natural Conditions

**DOI:** 10.1155/2013/308651

**Published:** 2013-07-17

**Authors:** Mohammad Moneruzzaman Khandaker, Golam Faruq, M. Motior Rahman, M. Sofian-Azirun, Amru Nasrulhaq Boyce

**Affiliations:** Institute of Biological Sciences, Faculty of Science, University of Malaya, 50603 Kuala Lumpur, Malaysia

## Abstract

Selected physiological and biochemical parameters were monitored at the vegetative and reproductive growth stages in potted *Bougainvillea* plants treated with five different concentrations of TRIA. Advanced flowering, flower bud number, and blooming rate increased significantly with 0.5 and 1.0 mg/L TRIA treatments. Similarly, photosynthetic rate, pigment content, quantum yield, and stomatal conductance increased significantly with 2.5, 1.0, and 5.0 mg/L TRIA treatments. Higher levels of N, P, and K, as well as increased total soluble solids (TSS) and higher sugar and protein contents, were recorded in treated plants. Furthermore, 46% more flowers, a 1.5-fold increase in bract weight, increased longevity, and 40% less leaf abscission were recorded following 2.5 mg/L TRIA treatment. Phenol and flavonoid contents, sucrose phosphate synthase (SPS), and antioxidant activities were also markedly increased with 2.5 and 1.0 mg/L TRIA treatments. However, ethylene production was significantly lower in the treated plants. Positive correlations were observed between leaf TSS and flowering time and flower number, between leaf sugar content and bract weight, and between net photosynthesis and bract growth and dry matter production. It can be concluded that the foliar spray of TRIA stimulates growth, enhances flowering, and improves the quality of potted *Bougainvillea* plants.

## 1. Introduction

*Bougainvillea *is a flowering ornamental plant that belongs to the family Nyctaginaceae (i.e., Four-O'Clock), which has 18 species and is an economically important ornamental flower in tropical and subtropical regions. The true, perfect flower is small, tubular, commonly white or yellow, and surrounded by showy, vibrantly colored petaloid bracts. The decorative quality of potted flowering plants depends on their flower and leaf number, bract color, longevity, and turnover. The commercial value of *Bougainvillea*, as for many potted plants, is seriously affected by the abscission of leaves and flowers, and this value can be improved by prolonging bract longevity and increasing its quality. In our previous study, it has been reported that *Bougainvillea* bracts are abscised within 4 to 5 weeks after blooming [1]. Darnell et al. [2] reported that flowering in many species can be induced by a variety of environmental techniques and the application of growth-promoting chemicals. 

Growth regulators play integral roles in controlling the growth, development, metabolism, and morphogenesis of flowering plants [3]. Gibberellic acid (GA_3_) was demonstrated to induce inflorescence development and flowering and to increase the number of flowers [4]. The application of kinetin and the removal of young leaves enhanced inflorescence development and improved the quality of *Bougainvillea *bracts [5]. In the present study, we tested the effects of TRIA, as well as other growth regulators, on the physiological activities and flowering behavior of *Bougainvillea* plants. TRIA, a saturated primary alcohol (n-C30H61OH), is a natural component of plant epicuticular waxes that can enhance plant growth [6, 7]. Many researchers have reported the positive role of TRIA in enhancing growth, yield, photosynthesis, nitrogen fixation, enzymatic activities, and levels of free amino acids, reducing sugars, and soluble protein [8]. TRIA application increased plant dry weight, protein and chlorophyll contents, and the net photosynthetic rate in rice [9]. It has also been reported that TRIA is involved in the upregulation of many genes that are involved in photosynthesis. Skogen et al. [10] reported that TRIA application increases plant growth, the number of inflorescences, and the quality of chrysanthemum (*Chrysanthemum morifolium*) flowers. Many researchers have examined the effects of TRIA on vegetables and certain agronomic crops, and these studies reported stimulatory effects on crop growth, yield, and quality. It is believed that the growth regulator TRIA could also have stimulatory effects on flowering plants. However, very few studies have examined how to improve the quality and longevity of *Bougainvillea *plants under natural or green house conditions. 

Currently, no information is available in the literature on the effects of TRIA on plant growth, flowering, and quality in potted *Bougainvillea *plants. This study evaluated the impacts of TRIA on improving the quality of *Bougainvillea *plants under natural conditions. It is proposed that TRIA application can improve the plant's physiological activities, stimulate flowering, and increase the quality of potted *Bougainvillea *plants. The findings of this study will provide a basis for future research into the growth-regulating effects of TRIA on *Bougainvillea *and other ornamental and flowering plants.

## 2. Materials and Methods

### 2.1. Experimental Site and Plant Material

The experiments were carried out at the Plant Physiology Garden at the Institute of Biological Sciences, Faculty of Science, University of Malaya, Kuala Lumpur, between 2010 and 2011. The experiments during the first season (June 2010–October 2010) and the second season (February 2011–June 2011) were performed in the same location. One-year-old seedlings of potted *Bougainvillea* plants were collected from a commercial nursery in Sungai Buloh, Selangor when the plants were 0.6 m long with approximately 6 to 8 secondary branches. These seedlings were planted in 7-inch pots filled with garden soil and peat soil in a ratio of 5 : 5. The plants were thoroughly watered when the soil appeared dry, approximately every 3 to 5 days, during the experimental period. All of the experiments, regardless of the season or year, were performed under the following normal prevailing conditions for this region: temperature 21–32°C, maximum PAR 2000 *μ*E m^−2^ s^−1^, and relative humidity of 60%–90%. Five grams of nutrients (N : P : K, at a ratio of 12 : 12 : 17) per plant were applied at 15-day intervals. Twenty *Bougainvillea *plants were used for each season. A completely randomized design (CRD) with four replications was used for each season's experiment.

### 2.2. Treatment Setting

A total of sixty uniform branches (three per plant) were selected for the treatment application in each season, and a single plant was taken as an experimental unit. Every branch was tagged 15 cm below the apex of the branch. The experiments consisted of five treatments (0, 0.5, 1.0, 2.5, and 5.0 mg/L TRIA), including the control, with four replications and three subreplications (12 replicates for each treatment). The treatments were applied at both the vegetative shoot and the reproductive stages, which exhibited strong growth with prolific and dense leaves. The selected branches were sprayed twice per week until inflorescence development. A total of ten spray applications were performed, five times before flowering and five times after flowering; 60 mL TRIA solution was used per treatment (twelve branches).

### 2.3. Growth Rate, Bract Length, Bract Weight, Blooming Rate, and Longevity of *Bougainvillea*

The leaf and shoot growth rates, flower bud number, blooming rate, bract length, and shoot elongation were measured at three-day intervals. Individual bract and flower weights, as well as bract, flower longevity, and leaf drops, were measured after 15 days of observation. All of the growth rates were measured using a vernier scale, and the growth per day (in cm) was calculated. Close observations were made to determine the number of nodes before the first inflorescence for each treatment. Individual bract and bract cluster weight, including the flowers, fresh biomass, and dry biomass, were measured using a Mettler PJ3000 balance, and bract lengths were measured on a Mitutoyo Vernier Scale. The dry matter content was measured in 0% moisture conditions. From the beginning of the experiments, 12 buds per treatment were selected for full blooming and longevity measurements. Three branches per treatment were selected for leaf abscission measurements. The observations were made when all of the bracts were open and abscission had occurred.

### 2.4. Mineral Content, Photosynthetic Pigment Levels, Quantum Yield, Photosynthetic Rate and Stomatal Conductance Measurements

The nutrient content of *Bougainvillea* leaves (N and P) was analyzed using a multielement analyzer (MEA). Grounded leaf samples were mixed with water, and 1 mL of the sample extract was injected into the MEA for the calculation. The potassium (K^+^) content of the leaves was determined using a Cardy potassium meter. The chlorophyll and carotene contents of the *Bougainvillea *leaves were determined using the methods described by Hendry and Price [11]. Chlorophyll fluorescence yield was measured using a Plant Efficiency Analyzer (Hansatech Instruments Ltd., England). Optimum quantum yield is presented as Fv/Fm, where Fv = relative variable fluorescence (Fm-Fo) and Fm = maximum fluorescence. The instrument was run at 27°C, with a time range of 10 *μ*s to 3 sec. The photosynthetic rate (Pn) of the *Bougainvillea* plants was measured using a portable photosynthesis system (Li-6400XT, Li-COR, USA), and the measurements were performed according to the methods described by Khandaker et al. [12]. Stomatal conductance was measured using a portable Porometer (Leaf Porometer, Model SC-1, USA). A leaf chamber was attached to one leaf and kept the leaf at an ambient temperature for 10–15 min to maintain sunlight adaptation. Stomatal conductance was measured in three replicates from different spots on a single leaf.

### 2.5. TSS, Total Sugar Levels, Protein Content, Phytochemical Constituents, SPS Activitys and Ethylene Production

The total soluble solids (TSS) content of the leaves was evaluated at 25°C with an Atago 8469 hand refractometer (Atago Co. LTD., Tokyo, Japan) and was expressed as °Bx. The total soluble sugar level of the leaves was determined using the phenol-sulphuric method [13]. Leaf protein content of the treated and nontreated branches was determined according to the Bradford method [14]. Antioxidant capacity was determined via the 2,2-diphenyl-1-picrylhydrazyl (DPPH) assay described by Tadolini et al. [15]. The total phenolic contents of the *Bougainvillea* leaves were determined using the Folin-Ciocalteu assay, as described by Singleton and Rossi [16]. The total flavonoid content was determined using the aluminum chloride colorimetric assay, using catechin as a standard [17]. Sucrose phosphate synthase activity was assayed under the Vmax condition according to the methods of Huber et al. [18]. The ethylene production of the *Bougainvillea* plants was measured during the vegetative and reproductive growth stages. Four leaves that were previously treated with TRIA were detached from four sample plants for each treatment stage, one at the vegetative growth stage and one more each on the 1st, 5th, and 10th days after flowering. The leaves were then incubated in a 25 mL sealed plastic jar for 2 h at temperatures ranging from 20°C to 28°C. Following the incubation, air samples were taken from the headspace for ethylene analysis using a Shimadzu GC-14A gas chromatograph. The ethylene production rates were calculated and expressed as nL g^-1 ^h^−1^. 

### 2.6. Statistical Analysis

A completely randomized design (CRD) with four replications (3 subreplications) was used for the treatments in both seasons' experiments. The data from both seasons were pooled and analyzed using MSTAT-C statistical software. The one-way ANOVA was applied to evaluate significant differences in the parameter studied for the different treatments. The least significant difference (LSD) and honestly significant difference (HSD) were calculated following a significant *F*-test (at *P* = 0.05).

## 3. Results

Table 1 shows the time course growth of *Bougainvillea* plants under natural conditions. TRIA treatments significantly affected the vegetative growth of *Bougainvillea *plants (Table 1). A faster growth rate was observed in leaf area and shoot length in all treatments when compared to the control group. The growth rate, as measured by shoot length, was 44% higher in the 2.5 mg/L TRIA-treated plant than in the control. This result was statistically significant, although no significant difference was observed for this parameter between the 2.5 mg/L group and the other TRIA treatment groups (Table 1). Shoot diameter growth was not significantly affected by TRIA treatments. The results indicated that flower bud and bract growth length were also significantly affected by TRIA treatments. Flower bud growth was higher in the 1.0, and 2.5 mg/L TRIA treatment groups than in the other treatment groups and the control group. This growth trend was observed during the entire reproductive period and resulted in a larger flower bud. The bract length growth rate was nearly 30% to 50% higher in all of the treatments when compared to the control group, and these differences between the treatments and the control were statistically significant (Table 1). Bract diameter growth was not significantly affected by TRIA treatments. 

**Figure 1:**
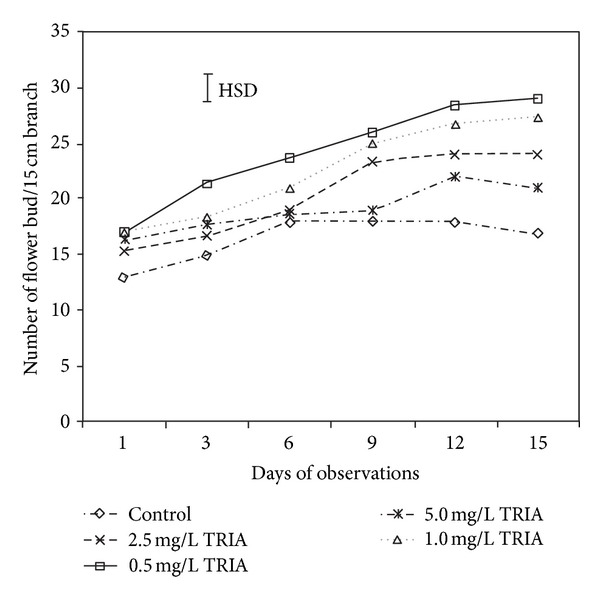
The effect of TRIA treatments on the flower bud formation of *Bougainvillea glabra* under natural conditions.

As shown by the results presented in Figure 1, TRIA treatments significantly increased the number of flower buds per 15 cm of branch. On the 12th day of observations, the flower bud number was 55%, 44%, and 35% higher in the 0.5, 1.0, and 2.5 mg/L TRIA treatment groups, respectively, compared to the control group. The most significant differences were observed when the branches were treated with 0.5 and 1.0 mg/L TRIA. This increasing trend in bud number was recorded throughout the entire observation period during the two seasons' experiments. 

**Figure 2:**
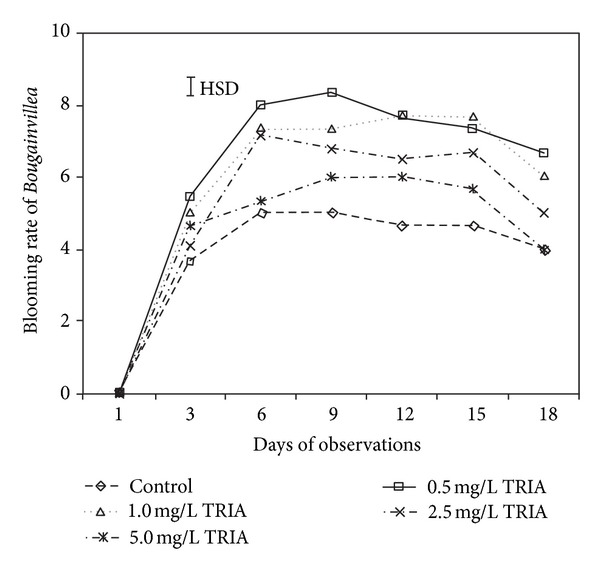
The effect of TRIA treatments on blooming rate of *Bougainvillea glabra* under natural conditions.

The results demonstrated that TRIA treatments significantly affected the bract blooming of *Bougainvillea *plants (Figure 2). It was observed that bract blooming increased for up to 2 weeks following bud development and thereafter decreased in the treated and untreated branches (Figure 2). By the 9th day of observation, the 0.5 mg/L TRIA treatment had produced the highest number of bract blooms (8) followed by the 1.0, 2.5, and 5.0 mg/L TRIA treatments, which resulted in 7, 7, and 5 bract blooms, respectively. The control plants produced the lowest number (3) of blooming bracts.

**Figure 3:**
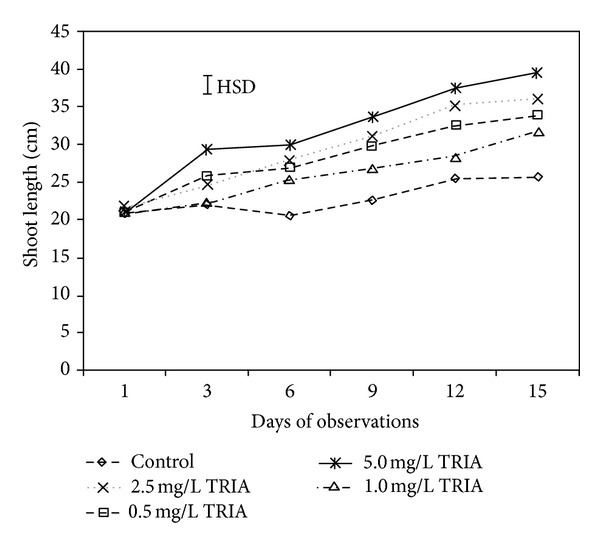
The effect of TRIA treatments on shoot length of *Bougainvillea glabra* under natural conditions.

The application of TRIA markedly increased the vegetative growth of *Bougainvillea* plants (Figure 3). The shoot growth rate in all of the treated plants was significantly higher throughout the experimental periods and was 32% higher in 5.0 mg/L TRIA-treated plants compared to the untreated group on the 3rd day of observations. It was also noted that the shoot growth rate increased with TRIA concentrations, and a relatively slow growth rate was observed in the control plants.

The accumulation of mineral content in the leaves of *Bougainvillea *was significantly affected by treatment with TRIA (Table 2). The results demonstrated that the 2.5 mg/L TRIA treatment resulted in the accumulation of 39%, 37%, and 35% more N, P, and K, respectively, when compared to control plants. The different treatments varied significantly among themselves only with respect to N accumulation in the leaves. TRIA treatment significantly affected the stomatal conductance of *Bougainvillea* leaves. The highest stomatal conductivity (38 m^2^s/mol) was observed in the 2.5 mg/L TRIA-treated leaves, followed by the other treatments and the control. The lowest stomatal conductivity (25 m^2^s/mol) was recorded in the control sample (Table 2). *Bougainvillea* plants that were treated with TRIA exhibited increased fresh and dry biomass production (Table 2). We found that the branches that were treated with 2.5 or 1.0 mg/L TRIA appeared to be healthier than those of the control plants and produced 1.28- or 1.23-fold more dry matter than the control plants, respectively. The next strongest effects were observed for the 5.0 and 0.5 mg/L TRIA treatments, and the branches from the control plants exhibited the lowest dry weights. 

Leaf chlorophyll content, which indirectly indicates the health status of a plant, was significantly affected by TRIA treatments in both seasons (Table 3). In the first season, the highest amounts of leaf chlorophyll *a*, *b*, and *a* + *b* content (2.7, 1.40, and 4.10 mg g^−1^ FW) were recorded for the 2.5 mg/L TRIA-treated plants followed by the other treatments and the untreated control group. In the second season, the 2.5 mg/L TRIA treatment also produced 25%, 22%, and 23% more chlorophyll *a*, *b*, and *a* + *b* contents, respectively, compared to the control plants. The 2.5 mg/L treatment was followed by the 1.0, 5, and 0.5 treatments, and the control plants exhibited the lowest chlorophyll contents. The carotenoid content of the leaves was also significantly affected by TRIA treatments (Table 3). The leaf carotenoid content was 26% and 30% higher in the 2.5 mg/L treated branches, compared to the control plants for the 1st and 2nd year observations, respectively. In all the treated plants, the leaf carotenoid content was higher in the treated than in the untreated plants, and the carotenoids in the leaves of *Bougainvillea* increased with flowering advance (data not shown). The photosystem of the leaves, as measured by quantum yield, had lower values in the nontreated plants; significantly higher quantum yield values were observed during the two years of study in all the TRIA-treated *Bougainvillea *plants compared to the untreated plants (Table 3).

**Figure 4:**
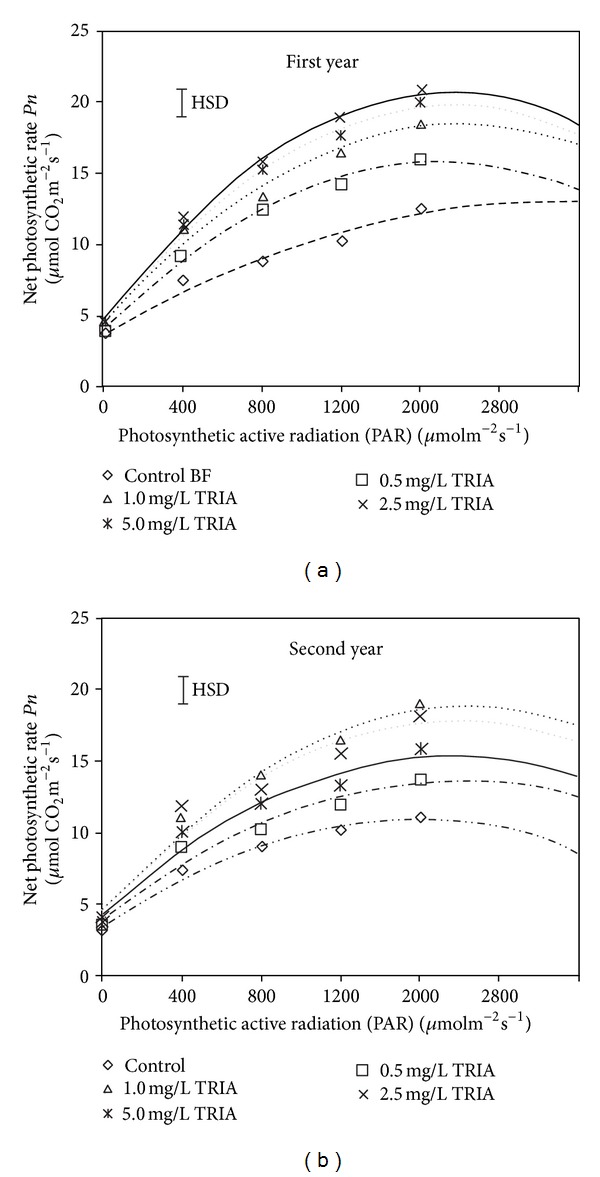
The effect of TRIA treatment on the net photosynthesis rate of *Bougainvillea glabra* under natural conditions.

The photosynthetic activity of *Bougainvillea* plants was determined, in terms of *μ*mol CO_2_ fixation m^−2^s^−1^, to measure the activity level of the photosynthetic carbon metabolism. In this study, TRIA treatments were observed to significantly increase the leaf photosynthetic activity in both growing seasons. In the first year, at 350 ppm CO_2_ and light intensities of 400, 800, 1200, and 2000 *μ*molm^−2^s^−1^, the photosynthetic activities were 1.6-, 1.7-, 1.8-, and 1.6-fold higher, respectively, in the 2.5 mg/L TRIA-treated leaves than in the control leaves (Figure 4(a)). Similarly, in the 2nd year, under the same conditions, the photosynthetic activities were 1.6-, 1.4-, 1.5-, and 1.6-fold higher, respectively, in the 2.5 mg/L TRIA-treated leaves (Figure 4(b)). It was also observed that the 2.5 and 1.0 mg/L TRIA-treated plants exhibited the highest photosynthetic activities in both seasons, while the control plants exhibited the least photosynthetic activity (Figures 4(a) and 4(b)).

Treatment with TRIA during the vegetative shoot stage resulted in an advanced flowering of the *Bougainvillea *plants. The results indicated that the 2.5, 5.0, and 1.0 mg/L treated branches produced flowers 12, 11, and 7 days earlier when compared to the untreated control branches, respectively. The differences between the treatment groups and the control group were statistically significant (Table 4). In the present study, it was observed that the TRIA-treated branches produced flowers following the appearance of approximately six fewer nodes compared to the control plants, and this was statistically significant between the treated and the control groups. Treatment with TRIA also increased the flower number significantly when compared to the control plants (Table 4). The highest number of flowers (13) was observed in a 2.5 mg/L treated branch, whereas the control branches produced the lowest number of flowers (7) per branch. As shown in Table 4, the TRIA treatments significantly increased the size and weight of *Bougainvillea* bracts, including the flowers. The highest bract weight (49 g), including the flower, was recorded in the 2.5 mg/L TRIA-treated plant, followed by the other treatments. The control branches exhibited the lowest bract and flower weights (approximately 39 g) (Table 4).

Foliar TRIA application increased the bract longevity significantly in *Bougainvillea* plants (Table 4). Our results indicated that treatment with 2.5 mg/L TRIA increased the bract longevity by approximately nine (9) days, followed by the 1.0 and 0.5 mg/L TRA treatments, which increased bract longevity by six (6) and four (4) days, respectively, compared to the control group. In this study, it was also observed that TRIA treatments at the vegetative shoot and reproductive stages reduced the leaf drop of *Bougainvillea*. Leaf drop was reduced by 40% in the plants treated with 2.5 mg/L TRIA, compared to the control plants, as observed 15 days after flowering (Table 4).

In this study, it was found that the TSS content of the *Bougainvillea *leaves significantly differed between the treated and the control leaves (Table 5). The highest TSS value (19.3°Bx) was observed in the branches treated with 2.5 mg/L TRIA, followed by the 1.0, 5.0 and 0.5 mg/L treatments. The lowest TSS value (16.5°Bx) was observed in the control branches. On the basis of two years of results, it was observed that TRIA application had a significant effect on the sugar content of *Bougainvillea *leaves. The leaf sugar content was 58% higher in the 2.5 mg/L TRIA-treated plants than in the control plants. The differences between the treatment and control groups were clearly significant (*P* < 0.05), whereas the differences in sugar content between the treatment groups were not statistically significant (Table 5). The same trend was also observed during the 2nd year (Table 5). Various concentrations of TRIA significantly affected the concentrations of phenols and flavonoids and the antioxidant activities of *Bougainvillea* plants (Table 5). Our results indicated that the 1.0 mg/L TRIA treatment yielded 1.15-, 1.14-, and 1.34-fold increases in the phenol and flavonoid contents and the antioxidant activities, respectively, compared to the untreated control group. The next strongest effects were observed for the 2.5, 5.0, and 0.5 mg/L TRIA treatments. The results were statistically significant between the treatment groups themselves and between the treatment and control groups. The soluble protein content of the *Bougainvillea* leaves was significantly affected by TRIA treatments (Table 5). The protein content was 18% higher in the 2.5 mg/L TRIA-treated leaves compared to the control group.

**Figure 5:**
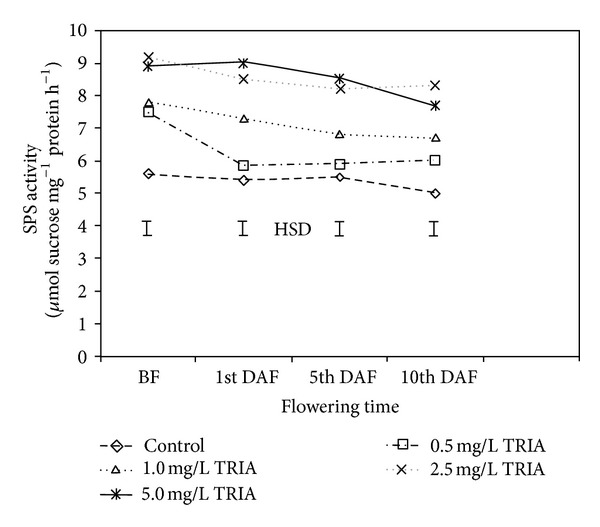
The effect of TRIA treatments on sucrose phosphate synthase (SPS) activity of *Bougainvillea glabra* leaves. BF: before flowering, DAF: day after flowering.

In the present study, the activity of sucrose phosphate synthase in the leaves of *Bougainvillea* plants was determined at the vegetative and reproductive growth stages. The results indicated that TRIA treatments significantly elevated the level and activity of the SPS enzyme in both growth stages (Figure 5). At the vegetative shoot stage, the SPS activity increased approximately 1.76-, 1.71-, 1.39- and 1.33-fold in the 2.5, 5, 1, and 0.5 mg/L TRIA treatment groups, respectively, compared to the control group. Accordingly, on the 1st, 5th, and 10th days of flowering, higher sucrose phosphate synthase activities were recorded in the treated plants then in the untreated group (Figure 5). It was also observed that SPS activities began to decrease with flowering time continued and that this trend was stronger in the control plants than in the treated plants.

**Figure 6:**
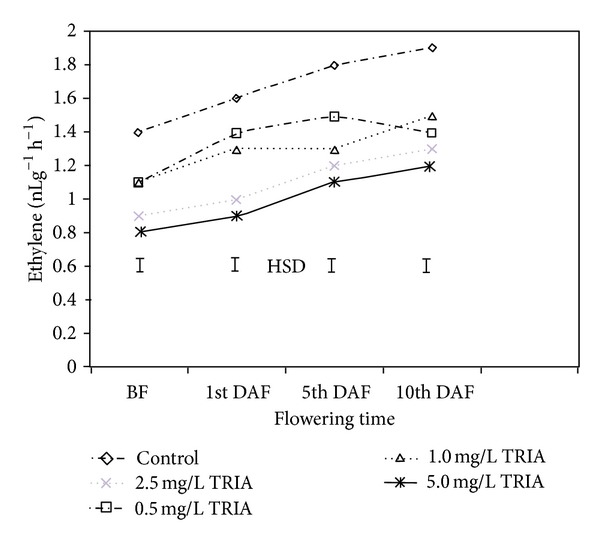
The effect of TRIA treatments on ethylene production of *Bougainvillea glabra* leaves. BF: before flowering, DAF: day after flowering.

Treatment with TRIA significantly reduced the ethylene production rate of *Bougainvillea* plants (Figure 6). During the vegetative shoot stage, the untreated leaves contained more ethylene (1.4 nLg^-1 ^h^−1^) than the treated plants, with the 2.5, 5, 1 and 0.5 mg/L treated leaves containing 0.8, 0.9, 1.1, and 1.1 nLg^-1 ^h^−1^ ethylene, respectively. During the 5th day of flowering, the ethylene production rate was 38%, 33%, 27%, and 16% lower in the 5, 2.5, 1, and 0.5 mg/L TRIA treatment groups, respectively, compared to the control group. It was also observed that the ethylene production rate increased with the flowering advance as well as with the age of the *Bougainvillea *plants, and the production rate was significantly lower in the treated plants compared to the control plants (Figure 6).

**Figure 7:**
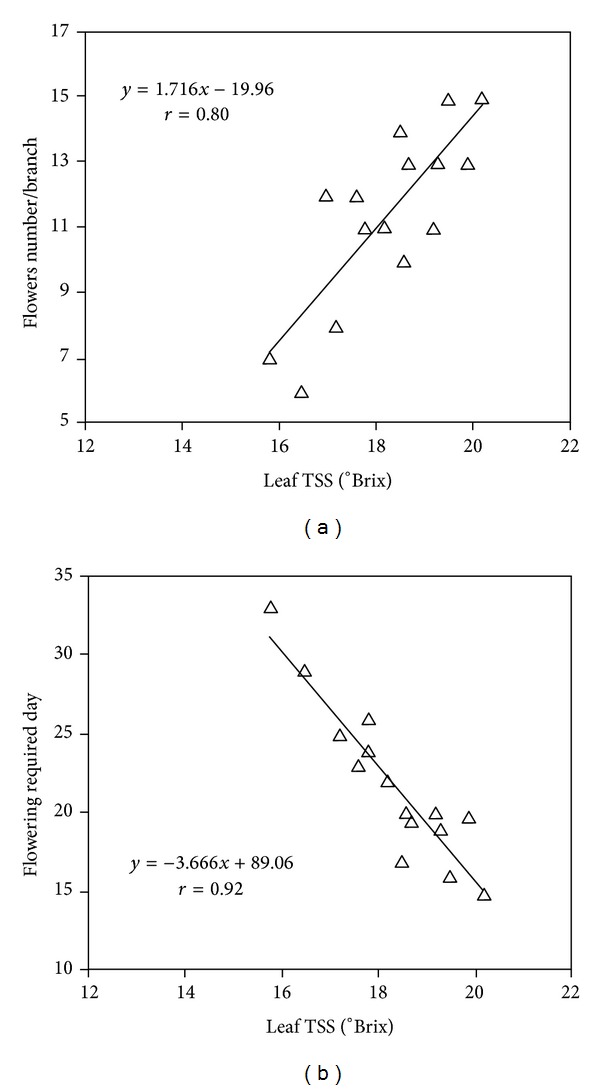
Regression lines for the relationship between flower number and leaf TSS (a) and flowering day and leaf TSS (b) in the treated *Bougainvillea* plants.

**Figure 8:**
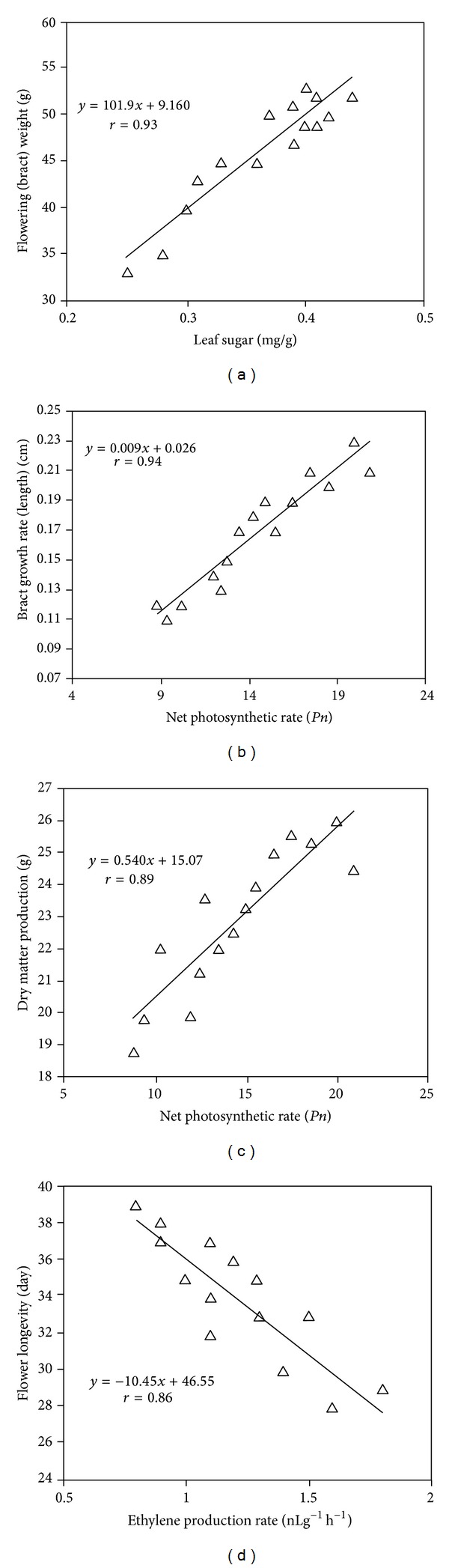
Regression lines for the correlations between flower weight (bract) and leaf sugar content (a), between net photosynthetic rate and bract growth rate (b), between net photosynthetic rate and dry matter production (c), and between flower longevity and the ethylene production rate (d) in TRIA-treated *Bougainvillea* plants under natural conditions.

In this two-year study, it was observed that several parameters were correlated in the TRIA-treated plants. A high degree of correlation was observed between the number of flowers and the leaf TSS (*r* = 0.80), as well as between the flowering required day and the leaf TSS (*r* = 0.92) (Figures 7(a) and 7(b)). Moreover, the flower weight (bract) was correlated with leaf sugar content (*r* = 0.93) as well as bract growth (*r* = 0.93), and dry matter production (*r* = 0.89) was positively correlated with the net photosynthesis rate of the treated plants (Figures 8(a), 8(b), and 8(c)). Furthermore, flower longevity was negatively correlated (*r* = 0.86) with ethylene production in *Bougainvillea* plants (Figure 8(d)).

## 4. Discussion

Triacontanol (TRIA) is a saturated long-chain alcohol that is known to have growth-promoting activities on a number of plants when applied exogenously. As an important growth regulator, TRIA has attracted much attention with respect to typifying its physiological effects on a number of agronomic crops. TRIA regulates various growth and developmental processes in plants under both normal and stress conditions [19]. Plants are sensitive to extremely low doses of TRIA; therefore, low concentrations of TRIA may be biologically active [20]. It has been reported that TRIA application enhances water uptake, cell division, cell elongation, and the permeability of plant cell membranes [21]. Our two-year study demonstrated that TRIA treatment increased leaf area, shoot length, flower bud, and bract growth of *Bougainvillea* plants. Reddy et al. [22] also reported that TRIA treatment increased the number of nodes, leaves, and shoots of* Capsicum frutescens *and *Decalepis hamiltonii*. Foliar application of 0.5 mg dm^−3^ TRIA significantly promoted the onset of flowering in green gram plants [23]. In this study, TRIA treatment stimulated bud formation and increased flower budding flower growth and the blooming rate of *Bougainvillea* plants. Skogen et al. [10] reported that TRIA treatment increased the growth, the number of inflorescences, and the quality of *Chrysanthemum morifolium *flowers. These authors also observed that the number of superior quality flowers was nearly doubled in the treated plants compared to control plants. TRIA may provide an active ingredient for bud formation, bud development, and the improved quality of flowers. Moreover, TRIA has been shown to increase the growth and/or yield of the majority of the major annual vegetables and agronomic crops as well as of forest species [24].

The results related to growth and biomass production of *Bougainvillea* plants are consistent with the findings of Muthuchelian et al. [25], who reported that TRIA treatments increased the root and shoot length, leaf density and area, and fresh and dry biomass accumulation of *Erythrina variegata* plants. These effects may be due to the rapid translocation of TRIA throughout the plant, causing a cascade of metabolic events and resulting in significant increases in growth and dry matter [26]. Naeem et al. [8] reported that foliar TRIA application significantly increased the N, P, K, and Ca contents of the leaves of hyacinth bean plants. The findings of the present study also demonstrate the significant effects on the accumulation of mineral content (N, P, and K) in *Bougainvillea* plants. Ries et al. [27] also reported that TRIA application stimulates K^+^, Ca^2+^, and Mg^2+^ accumulation in tomato, maize, and cucumber seedlings by eliciting a secondary messenger, L(^+^) adenosine. Elevated mineral contents may stimulate plant growth and flowering in *Bougainvillea* plants. It could also be suggested that the increased K^+^ content of the leaves may play a role in stomatal function.

Many investigators have explored the effects of TRIA on several basic metabolic processes, including photosynthesis, nutrient uptake, and enzymatic activities [8]. Photosynthetic capacity depends on photosynthetic pigments, such as chlorophylls *a* and *b*, and a salt-induced reduction in photosynthesis can be attributed to a decrease in chlorophyll content [28]. Here, it was reported that an accumulation of chlorophylls (*a*, *b*, and *a* + *b*) was significantly induced after foliar TRIA application. These results are similar to those reported by Muthuchelian et al. [25], who reported that TRIA treatment increased the synthesis of chlorophylls *a* and *b*, enhanced CO_2_ assimilation, and increased both the starch, and sugars content in *E. variegata* seedlings under different stress conditions. The observation of changes in the carotenoid content in leaves following TRIA applications is supported by the finding of Moorthy and Kathiresan [29], who reported that TRIA treatments increased the leaf carotenoid content of *Rhizophora apiculata*. The photosynthetic or quantum yield (Fv/Fm) of the treated plants was significantly affected by TRIA treatment in the present study. These quantum yield results are supported by the findings of Chen et al. [30], who stated that TRIA increased the minimal and maximal fluorescences (Fo and Fm) in rice plants. This effect might be due to TRIA increasing the antenna pigment level or the efficiency of excitation trapping at the active centers of PS-II. 

TRIA has a stimulatory effect on photosynthesis, and the increased growth and dry weight of plants treated with TRIA have been attributed to an improvement in photosynthesis and an enhanced accumulation of photosynthates [31]. Chen et al. [9] reported that higher transcription of the rbcS gene was associated with the improved photosynthetic activity in TRIA-treated plants. These authors also illustrated that TRIA affected photosynthesis by increasing the level and activity of ribulose-1,5-bisphosphate carboxylase oxygenase (RuBisCO) and by improving the status of the photosystems [31]. It has been demonstrated in a variety of plant species that the CO_2_ fixation rate increases when TRIA is applied in nanomolar concentrations [32]. In the present study, it was found that treatment with TRIA increased the net photosynthesis and stomatal conductance of *Bougainvillea *plants. Stomatal conductance affects the photosynthetic rate by regulating CO_2_ fixation in the leaf mesophyll tissue and is positively correlated with photosynthesis. Ivanov and Angelov [33] also reported that TRIA had positive effects on the net photosynthetic rate, stomatal conductance, and internal CO_2_ concentration of pea plants. The changes in branch dry matter that we observed are in agreement with the findings of Muthuchelian et al. [25], who reported that TRIA application increased the fresh and dry biomass of *Erythrina variegata* plants. In this present study, it was also observed that plant growth rate and dry matter production were positively correlated with the photosynthesis rate in the treated plants. A similar observation was reported by Eriksen et al. [31], who reported that dry matter production increased with the photosynthesis rate in maize.

The current study reported that TRIA treatments stimulated advanced flowering, reduced the node number required for the first flowering, and increased both the total flower number and the weight of flowers (bracts). These findings are supported by the results of Skogen et al. [10], who reported that TRIA treatment stimulates flowering, increases the growth and number of flowers, and improves the quality of chrysanthemum flowers. Ichimura et al. [34] reported that flower longevity was related to light intensity and the sugar supply to the sepals after flower opening. The present study demonstrated that TRIA treatment increased the flower longevity of *Bougainvillea *plants by delaying flower abscission. TRIA treatment may have increased the sugar supply to the leaf near the inflorescence and reduced the ethylene production by suppressing the activity of ACC synthase, thus increasing the longevity of the bracts and leaves. Moneruzzaman et al. [5] also reported that the sucrose application during postharvest storage increased the longevity of *Bougainvillea* bracts.

Ries [35] reported that TRIA resulted in simultaneous increases in soluble solids, soluble sugars and free amino acids in the treated plants. In this study, TRIA treatment also significantly increased the leaf TSS and sugar content of *Bougainvillea* plants. Houtz et al. [36] suggested that TRIA-stimulated an increase in the specific activity of the RuBisCO and phosphoenolpyruvate carboxylase enzymes. TRIA also increased the activity of a key respiratory enzyme, malate dehydrogenase, and other photosynthesis-related genes [37]. The elevated levels and activities of photosynthesis-related enzymes may have increased the TSS and sugar content of the leaves. The results also showed that the TSS content of leaves was positively correlated with advanced flowering and the increase in flower number of *Bougainvillea *plants. These findings are in agreement with those of Ramina et al. [38], who reported that higher TSS content of leaves stimulates flowering. In the current study, it was also observed that flower weight positively correlated with leaf sugar content in the TRIA-treated plants.

From this current study, it is clear that TRIA treatment produced a significant effect on both the phenol and flavonoid contents and the antioxidant activities of *Bougainvillea *plants. Similarly, Kumaravelu et al. [23] also reported that foliar TRIA application, at a dose of 0.5 mg dm^−3^, significantly elevated the saccharide, starch, amino acid, and phenol contents in green gram plants. TRIA may be responsible for the activation of PAL, chalcone synthase, and stilbene synthase gene expression. Grzegorczyk et al. [39] reported that TRIA treatment increased the biosynthesis of secondary metabolites and increased the antioxidant activities of *S. officinalis. *The results showed that the soluble protein content of *Bougainvillea *leaves increased significantly with TRIA treatment. These findings are similar to those of Chen et al. [9], who reported that TRIA application regulated ABA protein and downregulated the ABA gene in stressed rice plants. Kim et al. [40] also reported similar positive effects on the soluble protein, starch, sugar, and free amino acid contents in the leaves of *Oryza sativa* and *Zea mays.*

Enhanced sucrose phosphate synthase activity can be closely associated with an increase in sucrose accumulation in plant parts. The findings of this study demonstrated that TRIA application elevated the level and activity of the SPS enzyme during the vegetative and flowering growth stages of *Bougainvillea* plants. It was also observed that both the photosynthesis rate and SPS activity were positively correlated with TRIA treatment in *Bougainvillea* plants. This increasing rate of photosynthesis provides higher levels of phosphoglycerates (PGA), which in turn results in more fructose 6-phosphate and glucose 6-phosphate, increasing sucrose synthesis via SPS activity. The increase in sucrose accumulation may stimulate flowering, increase the number of flowers, enlarge the flower size, and delay abscission. Muthuchelian et al. [41] reported that the application of TRIA reduced the inhibition of PS-II and elevated the level and activity of RuBisCo in *E. variegate* plants under stress conditions. These authors also reported a TRIA-mediated maintenance in the photosynthetic machinery and a significant delay in the leaf senescence of water-stressed plants. In this study, it was also observed that TRIA significantly delayed leaf abscission in *Bougainvillea* plants. This effect may have been due to an increased accumulation of sugar in the treated leaves following TRIA treatment.

The ethylene biosynthetic enzymes 1-aminocyclopropane-1-carboxylic acid (ACC) synthase and ACC oxidase regulate ethylene synthesis and release in plants [42]. The constitutively synthesized ACC oxidase enzyme converts ACC to ethylene. Consequently, the accumulation of ACC augments ethylene production [43]. Here, we demonstrated that TRIA treatments led to a decline in ethylene production during both the vegetative shoot and flowering stages. The findings of this study are supported by the results of Saltveit and Dilley [44], who reported that TRIA in combination with either 10 mM kinetin or 10 *μ*M benzyladenine in pea plant extract prevented the reduction in wound ethylene synthesis. The observed decline in ethylene production during both stages is most likely due to a downregulation of the 1-aminocyclopropane-1-carboxylic acid (ACC) gene by TRIA. Ethylene production increased in both the treated and untreated plants over time. However, the ethylene production rate was slower in the TRIA-treated plants than in the untreated plants. This effect is most likely due to the slower conversion of ACC to ethylene. It was reported in this study that bract and leaf abscission is higher in the control plants compared to the TRIA-treated plants, an effect that may be due to the higher rate of ethylene formation in the untreated plants. Our results also suggested that flower longevity is negatively correlated with ethylene production rate in treated plants.

## 5. Conclusion 

The results have shown that the tested concentrations of TRIA used had a significant effect on photosynthesis, photosynthetic pigments, minerals, and biomass content of *Bougainvillea* plants. TRIA treatments also stimulated flowering and increased the number and quality of flowers in addition to increasing protein, TSS, and sugar content, as well as the antioxidant and SPS activities in *Bougainvillea* leaves. Ethylene production was markedly reduced whilst the longevity of the leaves and bracts was increased in TRIA-treated plants. It can be concluded that treatment with 1.0 and 2.5 mg/L TRIA can enhance the physiological activities, flowering, and quality of potted *Bougainvillea *plants grown under natural conditions. 

## Figures and Tables

**Table 1:** The effects of TRIA treatments on the growth rates of leaves, shoots, flower buds, and bracts of *Bougainvillea glabra* under natural conditions.

	Growth rate (cm/day): length and diameter							
Treatment (mg/L)	Leaf		Shoot		Flower bud		Bract	
	Length	Diam.	Length	Diam.	Length	Diam.	Length	Diam.
Control	0.12^b^	0.03^b^	0.16^b^	0.01^a^	0.19^d^	0.05^b^	0.12^c^	0.08^a^
TRIA 0.5	0.18^a^	0.04^b^	0.20^a^	0.01^a^	0.28^b^	0.07^a^	0.15^b^	0.10^a^
TRIA 1.0	0.20^a^	0.06^a^	0.21^a^	0.02^a^	0.38^a^	0.10^a^	0.18^a^	0.11^a^
TRIA 2.5	0.17^a^	0.08^a^	0.23^a^	0.02^a^	0.33^a^	0.12^a^	0.19^a^	0.11^a^
TRIA 5.0	0.19^a^	0.07^a^	0.21^a^	0.02^a^	0.25^c^	0.10^a^	0.17^b^	0.10^a^
LSD_0.05_	**0.06**	**0.05**	**0.05**	**ns**	**0.06**	**0.05**	**0.06**	**ns**

Values in a column sharing the same lower case letters are not significantly different at *P* < 0.05 (LSD test).

^a, b, c, d^Means within the same column followed by the same letter do not differ significantly according to the LSD test at *P* < 0.05; ns, not significant.

**Table 2:** The effects of TRIA treatments on the mineral content, stomatal conductance, and the fresh and dry biomasses of *Bougainvillea glabra* under natural conditions.

Treatment (mg/L)	Mineral ion content (mg/g)			Stomatal cond.	Fresh Wt of	Dry Wt of branch (g)
	N	P	k	(m^2^s/mol)	branch (g)	(dry matter)
Control	2.18^c^	0.29^b^	0.95^b^	25^c^	31.85^c^	19.85^d^
TRIA 0.5	2.80^b^	0.35^a^	1.20^a^	33^b^	34.30^b^	21.30^b^
TRIA 1.0	2.68^b^	0.38^a^	1.23^a^	34^a^	37.50^a^	24.50^a^
TRIA 2.5	3.06^a^	0.40^a^	1.29^a^	38^a^	39.30^a^	25.30^a^
TRIA 5.0	2.78^b^	0.35^a^	1.28^a^	37^a^	35.30^a^	22.50^a^
LSD_0.05_	**0.24**	**0.17**	**0.19**	**4.6**	**3.9**	**2.64 **

Values in a column sharing the same lower case letters are not significantly different at *P* < 0.05 (LSD test).

^a, b, c, d^Means within the same column followed by the same letter do not differ significantly according to the LSD test at *P* < 0.05.

**Table 3:** The effects of  TRIA on the photosynthetic pigments, carotenoids, and quantum yield of *Bougainvillea glabra* under natural conditions.

Treatment	Chl. *a*	Chl. *b*	Chl. *a* + *b*	Carotenoids	Quantum
(mg/L)	mg g^−1^ FW	mg g^−1^ FW	mg g^−1^ FW	mg g^−1^ FW	yield
July 10 to Oct/10					
Control	2.23^c^	1.18^d^	3.41^c^	1.03^c^	0.80^b^
TRIA 0.5	2.48^b^	1.30^b^	3.78^b^	1.20^b^	0.83^a^
TRIA 1.0	2.65^a^	1.39^a^	4.05^a^	1.35^a^	0.84^b^
TRIA 2.5	2.70^a^	1.40^a^	4.10^a^	1.30^a^	0.87^a^
TRIA 5.0	2.58^a^	1.26^c^	3.84^a^	1.28^a^	0.85^a^
LSD_0.05_	**0.15**	**0.09**	**0.28**	**0.10 **	**0.05**
Feb/11 to June/11					
Control	1.95^d^	1.05^c^	3.00^c^	1.08^c^	0.80^b^
TRIA 0.5	2.10^c^	1.25^a^	3.35^b^	1.27^b^	0.82^a^
TRIA 1.0	2.31^a^	1.32^a^	3.63^a^	1.39^a^	0.81^a^
TRIA 2.5	2.43^a^	1.28^a^	3.71^a^	1.40^a^	0.85^a^
TRIA 5.0	2.23^b^	1.20^b^	3.43^b^	1.27^a^	0.83^a^
LSD_0.05_	**0.13**	**0.08**	**0.14**	**0.08**	**0.06**

Values in a column sharing the same lower case letters are not significantly different at *P* < 0.05 (LSD test).

^a, b, c, d^Means within the same column followed by the same letter do not differ significantly according to the LSD test at *P* < 0.05.

**Table 4:** The effect of TRIA treatments on flowering initiation, quality, and the abscission of bracts and leaves of *Bougainvillea glabra* under natural conditions.

Treatment	Flowering	Node of first	Flowers	Fresh weight	Flower	Leaf drop
(mg/L)	required day	flower bud	per branch	of flowers	longevity	at 15 DAF
Control	29^a^	20^a^	7^c^	39^b^	29^c^	20^a^
TRIA 0.5	23^b^	14^b^	11^a^	45^a^	33^b^	15^b^
TRIA 1.0	22^b^	16^b^	12^a^	47^a^	35^a^	14^b^
TRIA 2.5	17^d^	14^b^	13^a^	49^a^	38^a^	12^b^
TRIA 5.0	18^c^	13^c^	10^b^	47^a^	35^a^	13^b^
LSD_0.05_	**4.7**	**2.4**	**2.3**	**6.8**	**3.2**	**3.8 **

Values in a column sharing the same lower case letters are not significantly different at *P* < 0.05 (LSD test).

^a, b, c, d^Means within the same column followed by the same letter do not differ significantly according to the LSD test at *P* < 0.05.

**Table 5:** The effects of TRIA on phytochemical levels and antioxidant activity in *Bougainvillea glabra* leaves.

Treatment	TSS	Total sugar		Phenol	Flavonoid	Antioxidant	Soluble
(mg/L)	(°Brix)	1st Y	2nd Y	(mg/g)	(mg/g)	(DPPH mg/g)	protein (mg/g)
Control	16.5^d^	0.28^b^	0.26^b^	2.94^c^	4.76^d^	223^b^	6.12^c^
TRIA 0.5	17.8^c^	0.33^a^	0.30^a^	3.18^b^	4.93^c^	258^b^	7.15^a^
TRIA 1.0	19.2^a^	0.39^a^	0.33^a^	3.39^a^	5.43^a^	300^a^	6.85^b^
TRIA 2.5	19.3^a^	0.42^a^	0.35^a^	3.26^a^	5.20^b^	312^a^	7.25^a^
TRIA 5.0	18.5^b^	0.40^a^	0.31^a^	3.30^a^	5.26^b^	264^b^	7.17^a^
LSD_0.05_	**1.40**	**0.5**	**0.5**	**0.14**	**0.08**	**41.4**	**0.34**

Values in a column sharing the same lower case letters are not significantly different at *P* < 0.05 (LSD test).

^a, b, c, d^Means within the same column followed by the same letter do not differ significantly according to the LSD test at *P* < 0.05.
